# Validity and item response theory properties of the Patient Health Questionnaire-9 for primary care depression screening in Mozambique (PHQ-9-MZ)

**DOI:** 10.1186/s12888-020-02772-0

**Published:** 2020-07-22

**Authors:** Vasco F. J. Cumbe, Alberto Muanido, Maria Nélia Manaca, Hélder Fumo, Pedro Chiruca, Leecreesha Hicks, Jair de Jesus Mari, Bradley H. Wagenaar

**Affiliations:** 1 Department of Mental Health, Sofala Provincial Health Directorate, Ministry of Health, Rua Poder Popular n.° 11 – 50. Caixa Postal 583. 4° Andar, Beira, Sofala Moçambique; 2 Departamento de Psiquiatria, Escola Paulista de Medicina, Universidade Federal de São Paulo, São Paulo, SP Brazil; 3 Faculdade de Medicina, Universidade Eduardo Mondlane, Maputo, Moçambique; 4 Health Alliance International, Beira, Mozambique; 5 Health Alliance International, Seattle, WA USA; 6 Department of Global Health, University of Washington, Seattle, WA USA; 7 Department of Epidemiology, University of Washington, Seattle, WA USA

**Keywords:** Validation, PHQ-9, Depression screening tool, Primary health care, Mozambique

## Abstract

**Background:**

Depression is one of the leading causes of disability in Mozambique; however, few patients with depression are identified in primary care. To our knowledge, there are no validated tools for depression screening in Mozambique. The aim of this study was to validate the Patient Health Questionnaire-9 (PHQ-9) for use in primary care settings in Mozambique.

**Methods:**

The PHQ-9 was adapted using a structured multi-phase process led by a team of bilingual experts followed by a review by lay individuals and pilot-testing including cognitive interviews. The final Mozambican PHQ-9 (PHQ-9-MZ) was applied among 502 individuals randomly selected from antenatal, postpartum, and general outpatient consultations in three Ministry of Health primary healthcare clinics in Sofala Province, Mozambique. The PHQ-9-MZ was evaluated against the MINI 5.0-MZ as a gold standard diagnostic tool.

**Results:**

The majority of participants were female (74%), with a mean age of 28. Using the MINI 5.0-MZ, 43 (9%) of the sample tested positive for major depressive disorder. Items of the PHQ-9-MZ showed good discrimination and factor loadings. One latent factor of depression explained 54% of the variance in scores. Questions 3 (sleep) and 5 (appetite) had the lowest item discrimination and factor loadings. The PHQ-9-MZ showed good internal consistency, with a Cronbach’s alpha of 0.84, and an area under the receiver operating characteristic curve (AUROC) of 0.81 (95% CI: 0.73, 0.89). The PHQ-2-MZ had an AUROC of 0.78 (95% CI: 0.70, 0.85). Using a cut-point of ≥9, the PHQ-9-MZ had a sensitivity of 46.5% and a specificity of 93.5%. Using a cut-point of ≥2, the PHQ-2-MZ had a sensitivity of 74.4% and a specificity of 71.7%. Increasing the cut-point to ≥3, the PHQ-2-MZ has a sensitivity of 32.6% and a specificity of 94.6%.

**Conclusions:**

The PHQ-9-MZ and PHQ-2-MZ emerge as two valid alternatives for screening for depression in primary health care settings in Mozambique. Depending on program needs and weighing the value of minimizing false positives and false negatives, the PHQ-9-MZ can be employed with cut-points ranging from ≥8 to ≥11, and the PHQ-2-MZ with cut-points ranging from ≥2 to ≥3.

## Introduction

The World Health Organization (WHO) recently reported that depression is now the leading cause of disability worldwide and that the number of individuals living with depression increased 18.4% between 2005 and 2015 [1] . Neuropsychiatric disorders, including depression, account for an estimated 14% of the global burden of disease [2]. Common mental disorders, including depression, have negative social, economic, and physical health impacts and are often co-morbid with other health problems such as HIV/AIDS and tuberculosis [3, 4]. The mortality risk for suicide among those living with depression is 20 times the general population, thus there is an urgent need for improved access to screening and care in areas with high rates of common mental disorders and poor access to mental health resources [5].

Mozambique has a great need for improved depression screening and care; the country is one of the poorest in the world, and its population has endured decades of trauma, from 1964 to 1975 due to anti-colonial struggle and a subsequent destabilization war from 1976 to 1992 leading to protracted political instability, war, displacement, and destruction of the public health system [6]. While there are limited population mental health measurements in Mozambique, data suggest the prevalence of severe mental illness is high (5.5%), especially in rural areas [7]. Models from the GBD consortium estimate that depression accounts for half of the YLD burden from MNS conditions in Mozambique and 10% of all YLDs nationwide [8]. A recent estimate by the WHO showed Mozambique to have an age-standardized suicide rate of 27.4/100,000, more than twice the global average of 11.4/100,000 and the highest suicide rate on the African continent [1]. Despite this significant demonstrated need for access, only 7.2% of primary care facilities offer mental health services in Mozambique and services are specialized and provided at the district level [3]. While psychiatric technicians and psychologists are co-located at these primary care facilities, the formal use of validated screening tools to screen primary care patients for common mental disorders, organized referral networks, or the integration between specialized psychiatric care and primary care are nascent. Mozambique has the conditions for rapid, large-scale changes in mental health coverage.

Over 90% of the population receives healthcare through the centralized public-sector Ministry of Health system of over 1300 public clinics [9]. The predominance of centralized public-sector clinics throughout the country suggests that pilot PHQ-9 validation results from primary care clinics in Sofala province may be generalizable to other public clinics across Mozambique. To our knowledge, no task-sharing and primary care intervention packages have been developed and tested in Mozambique, and few to none have been developed or tested in other Lusophone countries in Sub-Saharan Africa. The WHO Mental Health Gap Action Program (MhGAP) intervention guide was originally published in English in 2010, yet it was over 5 years before it was translated into Portuguese for use in Mozambique or other Lusophone countries [10].

Integrating depression screening and treatment into primary care is a growing focus of initiatives to improve access to mental health services, particularly in low- and middle-income countries (LMICs) [11]**.** The “integration of screening and core packages of services into routine primary health care” is one of the priority grand challenges to improve treatment and access to care for mental disorders globally [12]. This focus on integrating mental health into primary care has been advocated by leaders of the US National Institute of Mental Health [13], the World Health Organization [10] as well as African policymakers, researchers, and stakeholders [14]. Yet, many barriers to integration exist. For example, many health workers in LMICs are not trained and/or do not have time to screen for common mental disorders such as depression when seeing patients - a substantial barrier to integrating screening into routine practice and ensuring patients living with depression receive the care they need [15, 16]. Further, given limited financial and human resources, there is often limited availability of on-the-job training, re-training, and supervision for primary care staff in many LMICs. Brief, simple, culturally - relevant/adapted, and validated screening tools are thus an essential first step to efforts to integrate depression treatment into primary care.

Although a variety of screening tools for depression exist, few were developed specifically for use in LMIC settings; common mental disorders and their symptoms are often described or expressed in different manners depending on local contexts [15]. The Patient Health Questionnaire (PHQ-9 and PHQ-2) are short depression screening tools that are commonly used by health professionals in primary care settings in high-income settings [17]. The PHQ-9 was developed in the United States, and was validated with a sensitivity of 61% and specificity of 94% [18, 19]**.** However, variability in the performance of the PHQ-9 (and the short-form PHQ-2 version) across high-income contexts has been documented and is associated with inappropriate treatments and a potentially dangerous failure to identify depression in those patients needing treatment [17]. This variability in screening tool performance creates a particular challenge in LMICs such as Mozambique, where local idioms, low literacy rates, and different cultural conceptualizations of mental health can further reduce the usefulness of externally developed screening tools in detecting depression.

A 2016 systematic review of screening tools for common mental disorders in LMICs highlighted 20 existing validation studies of the PHQ-9 [15]. Of these, only four studies were in Sub-Saharan Africa, with one validation study each in Cameroon, Ethiopia, Uganda, and Nigeria. Yet, none of these studies were among primary care patients; two were among HIV+ patients attending regional treatment centers, one was among university students, and one was among patients at a large specialized referral hospital. Moreover, using cut-off scores of 10 there was very high variability in sensitivity (27–91%), specificity (77–99%), diagnostic odds ratio (6-10), and area under the receiver operating characteristic curve (AUROC); (0.68–1). An additional validation study of the PHQ-9 from South Africa showed that a cut-off of 9 achieved a sensitivity of 49% and specificity of 94%, with a diagnostic odds ratio of 14 and AUROC of 0.85 [20]. Another PHQ-9 validation study in Tanzania showed that an optimal cut-off of 9 achieved a sensitivity of 78% and specificity of 87% with a good overall accuracy (AUROC = 0.87) [21]. Last, a validation study in Malawi showed that using the optimal cut-point of ≥9, the PHQ-9 had a sensitivity of 64% and a specificity of 94% in detecting both minor and major depression, with high overall discrimination (AUROC = 0.93) [22];. The large variability in existing validation results using similar PHQ-9 cut-off points indicate a need to adapt and evaluate the performance of the PHQ-9 in diverse contexts prior to implementation.

The validation of the PHQ-9 in the primary care setting in Mozambique is one of the first steps towards creating contextually - relevant and effective tools to identify common mental disorders and in integrating depression care and treatment into primary care. This is an urgent need in Mozambique, as the existing specialized psychiatric care system focuses almost exclusively on severe mental illness, including schizophrenia and epilepsy. Initial pilot work has shown that less than 3% of all mental health consultations across Sofala Province and less than 2% of all consultations at the Beira Central Hospital were for any mood disorder [23, 24]. This suggests that a large burden of depression is missed in existing psychiatric care settings [25]. A recent population-based survey of over 3000 households conducted by our group additionally showed the high rates of mental health stigma in central Mozambique, as well as treatment gaps for depressive symptoms and suicidal ideation of almost 70 and 90%, respectively [26, 27].

This study aimed to develop an initial Mozambican adaptation of the PHQ-9 (PHQ-9-MZ) and to test the validity and initial item response theory properties of the PHQ-9 and the PHQ-2 among primary care patients. To our knowledge, this is the first study to validate a depression screening tool for use in Mozambique. We hope these findings can inform future tool development for mental health assessment in Mozambique and drive forward improvements in closing the gap for treatment of common mental disorders. We also anticipate these findings to be of interest to investigators in other similar LMICs, and especially Lusophone African countries.

## Methods

### Structure of mental health system in Mozambique

The National Mental Health Program in Mozambique is managed by the Department of Mental Health at the National Directorate of Public Health in the Ministry of Health. At the Provincial Health Directorate, the Provincial Mental Health Program is part of the Department of Public Health and is responsible for coordinating all mental health activities implemented in the districts and reporting to the National Mental Health Program in the capital of Maputo, Mozambique. At the district level, the District Mental Health Supervisor coordinates and supports activities implemented at health facilities and reports these activities to Provincial Mental Health Program. The country has 25 psychiatrists (18 of which are Mozambican), 305 psychiatric technicians, 130 clinical psychologists, and 14 occupational therapists who provide services to an estimated 7% of public clinics. Since 1996, Mozambique has been a leader in Sub-Saharan Africa in training a task-shared cadre of mental health professionals (Psychiatric Technicians) who can diagnose and treat all major categories of mental illness, with a focus on psychopharmacology. In 2014, the Mozambican Ministry of Health accomplished their goal of placing at least one psychiatric technician at a primary care health facility within each of the 135 districts nationally [14, 23]; however, the vast majority of psychiatrists are located in the capital of Maputo, Mozambique.

### Study setting and participants

**Fig. 1:**
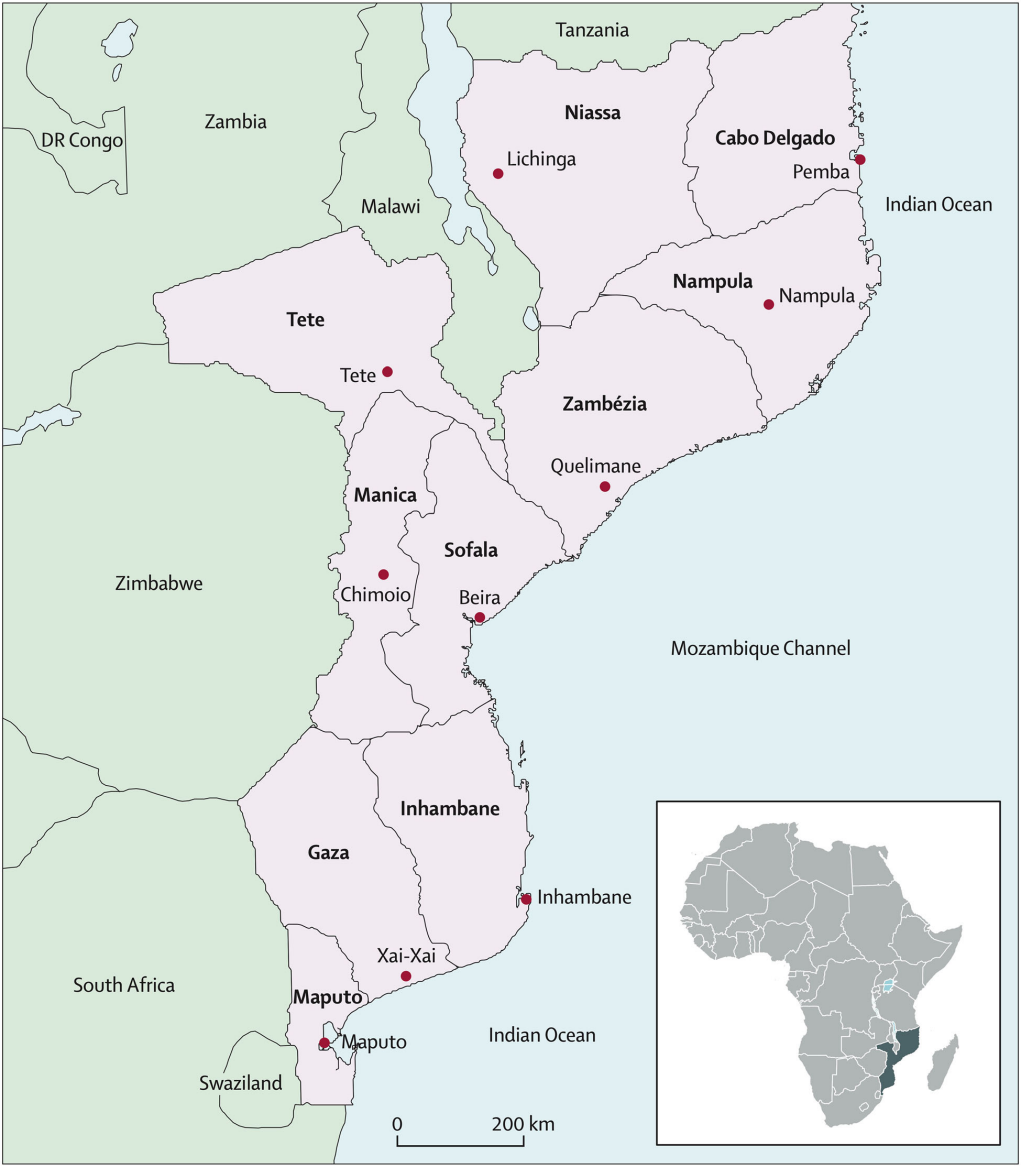
Political map of Mozambique including provincial capital cities. Focal area of Beira City and surrounding Dondo highlighted by the red box. Figure sourced from: Fernandes QF, Wagenaar BH, Anselmi L, Pfeiffer J, Gloyd S, Sherr K. Effects of health-system strengthening on under-5, infant, and neonatal mortality: 11 year provincial-level time-series analyses in Mozambique. Lancet Global Health. 2014; 2 [8]

This study was conducted in Sofala Province, (see Fig. 1), located in the central region of Mozambique with a population of approximately 2.2 million. The official language is Portuguese, with ​​Cisena and Cindau common languages spoken in rural areas. Sofala has a literacy rate of 56.4%, infant mortality of 83.3 per 1000 live births, life expectancy of 50 years, and an HIV prevalence of 14% [28]. As a whole, Sofala province has 166 health facilities, of which 25 (15%) have trained mental health staff. These staff include 3 Psychiatrists, 29 Clinical Psychologists, 28 Psychiatric Technicians and 1 Social Worker [29]. The present study was conducted in 3 health facilities: 2 in Beira City (Macurungo and Chingussura), and 1 in Dondo (Dondo health facility). Beira is the capital of Sofala Province and the second largest city in Mozambique after the national capital of Maputo. Beira City has a population of approximately 500,000 individuals. Regarding health infrastructure, Beira City has 13 primary care health facilities, 1 quaternary-level central hospital, and several private health facilities. Dondo is the closest city to Beira (35 km), with 8 primary care health facilities serving a population of 91,000 [28]. We selected the above-mentioned facilities because they: [1] had at least 1 psychiatric technician and clinical psychologist [2]; were high-flow facilities providing general primary healthcare [3]; provided comprehensive maternal and child healthcare; and [4] were generally representative of other urban and peri-urban primary care health facilities in Mozambique.

### Adaptation of PHQ-9 to the Mozambican context (PHQ-9-MZ)

The PHQ-9 is a self-administered nine-item screening tool for depression that refers to the past 2 weeks with likert scale responses of how often a person has been bothered by symptoms, including “0 = not at all”, “1 = several days”, “2 = more than half of the days”, and “3 = nearly every day”. This tool can be used to screen for depression in at-risk populations and to monitor the severity of depression and treatment response [18, 30]**.** The adaptation and creation of the PHQ-9-MZ, from the original English version [18], occurred from February 2016 to April 2016 with a structured process to ensure content, semantic, and technical equivalence. This focused initially on a series of translations of the English PHQ-9, with a focus on comprehensibility (does an item retain its original semantic equivalence), appropriateness (fit, relevance, compatibility with new cultural context), and a specific focus on ease-of-understanding given the low literacy levels of primary care patients in Mozambique. We followed a modified version of the WHO’s seven steps for the translation and adaptation process [31], with the addition of cognitive interviewing of primary care patients after the first PHQ-9 translation. First, we established a bilingual group of experts, including a local Mozambican psychiatrist (VFJC), an American psychiatric epidemiologist (BHW), and an experienced local Mozambican psychiatric technician (HF). Second, this group examined and discussed the structure of the English PHQ-9. Third, this group collaboratively translated each PHQ-9 item. Fourth, this group examined the translation and refined initial elements. Fifth, the group (led by HF and a second psychiatric technician blinded to the original instrument (PC)) administered the PHQ-9 to 12 primary care patients attending outpatient consultations at Beira Central Hospital, Ponta-Gêa, and Munhava health facilities in Beira City, Mozambique. After administering the PHQ-9, HF and PC guided patients in a cognitive interview process whereby they asked patients what they felt was the underlying significance of each question, whether the question was unclear or inappropriate, and if so, how they might suggest improving each question. Sixth, following cognitive interviews, VFJC, BHW, HF, and PC reviewed the cognitive interviewing data and engaged in a collaborative process of improving the instrument based on this feedback. Last, HF and PC again pilot-tested the final instrument amongst 4 local Mozambican health staff of various literacy working at Health Alliance International, all of whom found the adapted PHQ-9-MZ instrument comprehensible, appropriate, and easy to understand.

### Adaptation of the MINI international neuropsychiatric interview to the Mozambican context (MINI 5.0-MZ)

The MINI International Neuropsychiatric Interview 5.0 (MINI 5.0) is a short structured diagnostic interview developed in the United States for Diagnostic and Statistical Manual for Mental Disorders 4 (DSM-IV) and International Classification of Diseases 10 (ICD-10): Psychiatric Disorders. It is administered approximately in 15 min [32]. The MINI includes a structured psychiatric interview for all common mental disorders and was used in this study as a gold standard diagnostic tool to validate the PHQ-9. In this study, the administration of the MINI 5.0-MZ took an average of 30–45 min.

For this study, we adapted the existing Brazilian Portuguese version of the MINI 5.0 to the Mozambican context (MINI 5.0-MZ). Following a similar method as the adaptation of the PHQ-9, we first recruited a group of local Mozambican mental health professionals (2 clinical psychologists and 3 psychiatric technicians) to collaboratively adapt the Brazilian MINI 5.0 to the Mozambican context and linguistic idioms. Second, the instrument was coded in RedCap for use on tablets by a local Mozambican study staff member (AM). Following coding, the same group of mental health staff re-reviewed the MINI 5.0-MZ in RedCap and focused on understanding, ease of use, and logical sequence of questions. Fourth, mental health professionals from each target health facility had a 2-day training for 2 to 3 h a day on the correct use of the MINI 5.0-MZ. Fifth, mental health staff conducted role plays where one professional was a patient and the other administered the MINI 5.0-MZ, with notes taken and reviewed for instrument improvement. Sixth, the MINI 5.0-MZ was pilot-tested over 4 days among 14 primary care patients attending outpatient consultations at Macurungo heath facility in Beira City. Seventh, patients administered the pilot MINI 5.0-MZ were guided in a cognitive interview process whereby they were asked what they felt was the underlying significance of each question, whether the question was unclear or inappropriate, and if so, how they might suggest improving each question. Following cognitive interviews and the pilot implementation, the mental health professionals, along with VFJC, BHW, and AM reviewed the cognitive interview data and engaged in a collaborative process of improving the MINI 5.0-MZ based on this feedback.

### Data collection procedures

From October 2018 to February 2019, two trained data collectors, supervised by AM, administered a survey using tablet-based RedCap data collection that included sociodemographic variables and the PHQ-9-MZ, to 502 randomly selected patients from the waiting room of antenatal, postpartum, and general outpatient consultations. While in the waiting room, a data collector randomly selected individuals and asked them if they would be willing to complete a survey on depression. The data collector then directed interested individuals to a private room to administer the survey if they were 18 years old or over and agreed to participate in the study by signing an informed consent form. Patients were excluded if they had an acute health condition or disability impeding their ability to complete the survey. This initial survey took approximately 30–40 min. Following this survey, patients were referred to the trained mental health professional (psychologist or psychiatric technician) who administered the MINI 5.0-MZ, as the gold standard diagnostic validation tool, blinded to the responses of the patient on the PHQ-9-MZ.

### Data analysis procedures

Using Stata 15 we calculated the sensitivity, specificity, positive predictive values, negative predictive values, and diagnostic odds ratios across screening cut-points for the PHQ-9-MZ and PHQ-2-MZ, using the MINI 5.0-MZ as gold standard. The receiver operating characteristic curves were graphically examined and the area under the ROC (AUROC) was calculated for each instrument. To examine initial item response theory properties of the PHQ-9-MZ, the item discrimination (α; describing how well a given item can differentiate between patients with different levels of depressive symptoms), item location (*b*_*1*_*; b*_*2*_*; b*_*3*_; the level of the latent trait of depression where the probability of endorsing a given item is 50%), item factor loadings, and item uniqueness were calculated. The item information functions, the full PHQ-9-MZ test information function and standard error, and test characteristic curve and expected scores for different values of the latent trait of depression were also graphically examined. Last, the Cronbach’s alpha [33], including item-test correlations, item-rest correlations, average inter-item covariances, and the Cronbach’s alpha value if each item were to be removed individually were calculated.

## Results

### Results of the adaptation process for the PHQ-9-MZ

During the adaptation process, a few questions from the English PHQ-9 did not easily translate to the Mozambican context. Specific difficulties included Question 6, with the original PHQ-9 reading: *“Feeling bad about yourself - or that you are a failure or have let yourself or your family down”.* After initial direct translations into Mozambican Portuguese, primary care patients had trouble understanding the concepts that the question was intending to cover. The final Question 6 in the PHQ-9-MZ directly translated into English reads: “*Feeling like you do not like yourself, that you are a failure / not useful / have no worth or that you let yourself or your family down”.* Specific efforts were also directed at adapting Question 7, with the original PHQ-9 reading: *“Have trouble concentrating on things, such as reading the newspaper or watching television”.* The activity of reading the newspaper was deemed inappropriate for screening low-literacy populations common in Mozambique. In addition, many rural or poorer individuals do not commonly have a television available. The final Question 7 on the PHQ-9-MZ directly translated into English reads: *“Have a lack of concentration in doing things, such as working, studying, home chores, or other activities.* Last, in contrast to the original English PHQ-9, the PHQ-9-MZ included: *“In the last two weeks, how many days have you…”* as the beginning to each question, rather than referring to this time period generally in the introduction. This was to facilitate understanding and comprehension when the PHQ-9-MZ is administered orally to patients (see Table 1 for detailed PHQ-9-MZ).

**Table 1:** Adaptation and translation of English version of Patient Health Questionnaire 9 for use in primary care settings in Mozambique (PHQ-9-MZ)

Item	Mozambican Portuguese version
PHQ1-MZ	Nas últimas 2 semanas, quantos dias você teve pouco interesse ou prazer em fazer as coisas que você gostava.
PHQ2-MZ	Nas últimas 2 semanas, quantos dias você sentiu-se em baixo, triste ou desesperado.
PHQ3-MZ	Nas últimas 2 semanas, quantos dias você teve dificuldades de apanhar sono, manter o sono, ou dormir muito ou pouco tempo.
PHQ4-MZ	Nas últimas 2 semanas, quantos dias você sentiu - se cansado / com pouca força ou com pouca energia.
PHQ5-MZ	Nas últimas 2 semanas, quantos dias você teve falta de apetite ou comer muito.
PHQ6-MZ	Nas últimas 2 semanas, quantos dias você sentiu que não gosta de si próprio/a, ou que é fracassado / inútil / não serve para nada ou que tem deixado a si e a sua família para baixo.
PHQ7-MZ	Nas últimas 2 semanas, quantos dias você teve falta de concentração em fazer as coisas, como trabalhar, estudar, trabalhos domésticos, ou outras actividades.
PHQ8-MZ	Nas últimas 2 semanas, quantos dias você esteve a falar, agir, ou mover-se lentamente, ou ficar irrequieto ou agitado do que o habitual ou que outras pessoas terão notado.
PHQ9-MZ	Nas últimas 2 semanas, quantos dias você pensou que seria melhor morrer ou fazer mal a si mesmo.
English response 1: Not at all	Nunca (0 dias)
English response 2: Several days	Algumas vezes (1–7 dias)
English response 3: More than half the days	Muitas vezes (8–11 dias)
English response 4: Nearly every day	Todos ou quase todos os dias (12–14 dias)

### Sociodemographic characteristics

As described in Table 2, the majority of the 502 randomly selected primary care patients were female (74%), with a mean age of 28 (SD = 7.4). The majority of patients (66%) were in the age group of 18 to 29 years old. Forty-five percent (*n* = 224) of patients were recruited from outpatient primary care, 28% (*n* = 140) from prenatal care, and 28% (*n* = 138) from post-partum care. The majority were in a civil union (71%, *n* = 355). More than half of the patients had either completed high-school or some high-school. The average number of people living in patients’ household was 5. Patients earned a mean of $117 United States Dollars per month. Almost one-third of the sample was HIV+ (28%), with 5% having never been tested for HIV (see Table 2 for more information).

**Table 2:** Demographic characteristics of 502 patients administered Mozambique-adapted Patient Health Questionnaire-9 (PHQ-9-MZ) and MINI International Neuropsychiatric Interview (MINI 5.0-MZ) in primary care settings in Sofala, Mozambique

Characteristic	Total (N, %), unless noted	Positive on MINI 5.0-MZ for Major Depression (N, %), unless noted	Negative on MINI 5.0-MZ for Major Depression (N, %), unless noted
**Total**	502 (100)	43 (8.6)	459 (91.4)
**Age, Mean (SD)**	**27.8 (7.4)**	**26.9 (6.0)**	**27.9 (7.5)**
Age 18–24	190 (37.9)	16 (37.2)	174 (37.9)
Age 25–29	142 (28.3)	15 (34.9)	127 (27.7)
Age 30–34	63 (12.6)	5 (11.6)	58 (12.6)
Age 35+	96 (19.1)	7 (16.3)	89 (19.4)
Don’t know	11 (2.2)	0 (0)	11 (2.4)
**Sex**			
Male	130 (25.9)	13 (30.2)	117 (25.4)
Female	372 (74.1)	30 (69.8)	343 (74.6)
**Purpose of visit**			
Prenatal Consultation	140 (27.9)	9 (20.9)	131 (28.5)
Post-partum Consultation	138 (27.5)	8 (18.6)	130 (28.3)
Outpatient Primary Care	224 (44.6)	26 (60.5)	198 (43.1)
**Marital status**			
Single	100 (19.9)	8 (18.6)	92 (20.0)
Married	29 (5.8)	4 (9.3)	25 (5.5)
Civil Union	355 (70.7)	26 (60.5)	329 (71.7)
Separated	7 (1.4)	3 (7.0)	4 (0.87)
Divorced	4 (0.80)	1 (2.3)	3 (0.65)
Widowed	7 (1.4)	1 (2.3)	6 (1.3)
**Education level**			
No school	2 (0.40)	0 (0)	2 (0.44)
Some Primary	74 (14.7)	8 (18.6)	66 (14.4)
Finished Primary	56 (11.2)	9 (20.9)	47 (10.2)
Some High School	157 (31.3)	10 (23.3)	147 (32.0)
Finished High School	138 (27.5)	9 (20.9)	129 (28.1)
Post-Graduate	65 (13.0)	7 (16.3)	58 (12.6)
Missing	10 (2.0)	0 (0)	10 (2.2)
**# living in house, Mean (SD)**	**5.0 (2.3)**	**5.0 (2.6)**	**5.0 (2.3)**
1–3	137 (27.3)	14 (32.6)	123 (26.8)
4–6	257 (51.2)	20 (46.5)	237 (51.6)
7–9	92 (18.3)	6 (14.0)	86 (18.7)
10+	16 (3.2)	3 (7.0)	13 (2.8)
^**a**^**USD earned per month, Mean (SD)**	**116.6 (145.4)**	**160.7 (294.3)**	**112.6 (123.4)**
Don’t know	57 (11.2)	6 (14.0)	50 (10.9)
**HIV+**			
Yes	140 (27.9)	12 (27.9)	128 (27.9)
No	335 (66.7)	28 (65.1)	307 (66.9)
Never tested	27 (5.4)	3 (7.0)	24 (5.2)

^a^Converted to USD using current exchange rate which is 61 MZN to one USD

### Major depressive disorder characteristics

The MINI 5.0-MZ tested positive for major depressive disorder (MDD) among 43 patients – 9% of the overall sample. No statistical testing of associated sociodemographic factors and depression was conducted; however, individuals testing positive for MDD appeared younger, were more likely to be recruited from outpatient care, and had higher monthly income than individuals testing negative for MDD. There were no appreciative differences in prevalence of MDD by HIV status (see Table 2 for more information).

### Item response theory properties of the PHQ-9-MZ

**Fig. 2:**
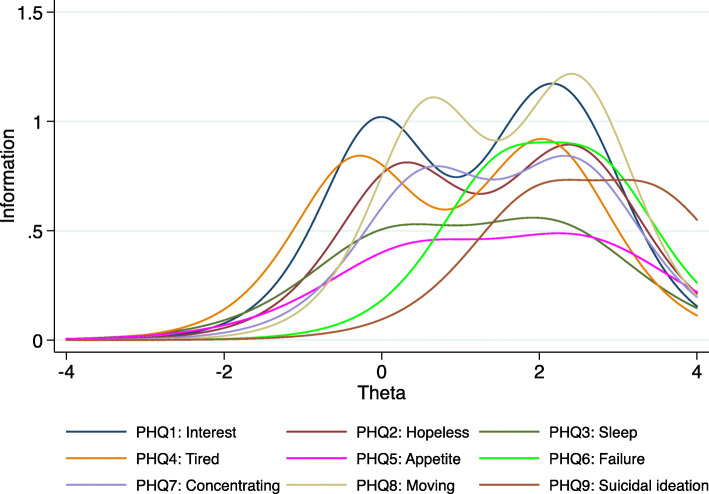
Item information functions from item response theory graded response model for Patient Health Questionnaire 9-item (PHQ-9) screening tool among primary care patients in Mozambique

**Fig. 3:**
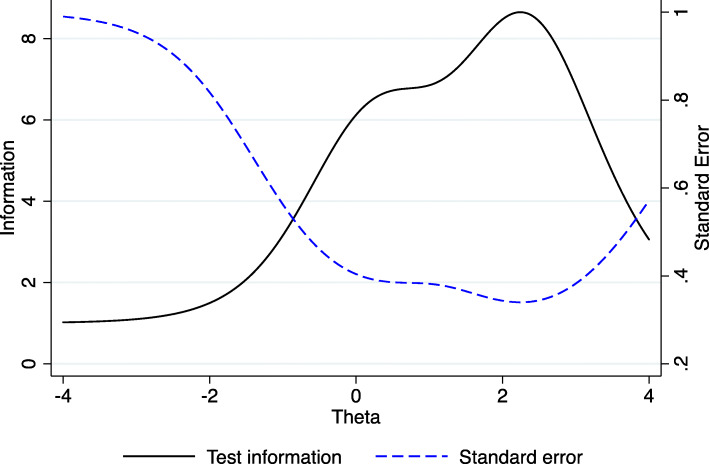
Test information function and standard error for the Patient Health Questionnaire 9-item (PHQ-9) screening tool among primary care patients in Mozambique

**Fig. 4:**
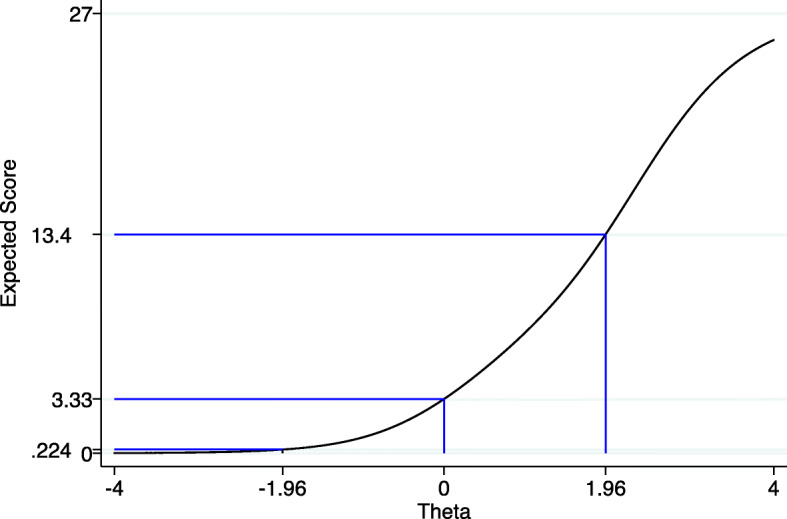
Test characteristic curve and expected scores for different values of the latent trait (Theta) for the Mozambican Patient Health Questionnaire 9-item (PHQ-9-MZ) screening tool among primary care patients in Mozambique

Individual PHQ-9-MZ item discrimination (α) ranged from 1.3 to 2.1, indicating a moderate variation in item discrimination (see Table 3). Item discrimination (α) determines how well a question can discriminate between individuals with high versus low depressive symptoms. Question 8 on movement/agitation (α = 2.1) had the strongest discrimination, followed by question 1 on loss of interest (α = 2.0). Question 5 on loss of appetite had the weakest discrimination (α = 1.3), followed by question 3 on sleep (α = 1.4). The sub-optimal performance of questions 5 and 3 can be easily visualized by the flat nature of the item information functions in Fig. 2. Question 9 was the most “difficult” to endorse, in that it was endorsed only at the highest trait levels (b); (see Table 3 and Fig. 2). Following this question, the second most difficult question to endorse was question 6 on failure. Figure 3 displays the test information function for the PHQ-9-MZ, showing maximum information and minimum standard error at approximately 2 standard deviations above the mean depression trait levels. Figure 4 displays the test characteristic curve for the PHQ-9-MZ, showing that at 1.96 standard deviations above the mean depression trait levels, individuals score an average of 13.4 on the PHQ-9-MZ. Individuals with average sample depression levels (Theta = 0) score 3.3 on the PHQ-9-MZ. Individuals 1.96 standard deviations below mean depression trait levels score an average of 0.24 on the instrument.

**Table 3:** Item Response Theory Properties, Factor Loadings, and Cronbach’s alpha of Patient Health Questionnaire 9-item (PHQ-9) and Patient Health Questionnaire 2-item (PHQ-2) Screening tools Administered in Primary Care Settings in Sofala, Mozambique

Item	IRT Parameters				Factor loadings	Uniqueness	Item test correlation	Item rest correlation	Average interitem covariance	Item Cronbach’s Alpha
	α	***b***_***1***_	***b***_***2***_	***b***_***3***_						
PHQ1: Interest	2.00	−0.04	1.94	2.41	0.722	0.479	0.719	0.619	0.177	0.814
PHQ2: Hopeless	1.78	0.25	2.26	2.57	0.717	0.486	0.690	0.583	0.181	0.818
PHQ3: Sleep	1.40	0.12	1.94	2.25	0.582	0.661	0.620	0.466	0.182	0.835
PHQ4: Tired	1.83	−0.31	1.93	2.17	0.701	0.508	0.720	0.611	0.174	0.815
PHQ5: Appetite	1.29	0.40	2.25	2.69	0.564	0.682	0.629	0.490	0.183	0.830
PHQ6: Failure	1.78	1.53	N/R	2.71	0.747	0.443	0.651	0.558	0.191	0.822
PHQ7: Concentrating	1.74	0.56	2.31	2.51	0.728	0.469	0.712	0.610	0.178	0.815
PHQ8: Moving	2.08	0.60	2.32	2.57	0.753	0.433	0.718	0.627	0.181	0.814
PHQ9: Suicidal ideation	1.63	2.0	N/R	3.44	0.737	0.457	0.535	0.456	0.209	0.833
**Overall PHQ-9**									**0.184**	**0.839**
**Overall PHQ-2**										**0.606**

Abbreviations: *IRT* Item Response Theory, *N/R* No responses at this level; α = item discrimination; *b*_*1*_ refers to endorsing “several days”, Portuguese: “Algumas vezes”; *b*_*2*_ refers to endorsing “more than half the days”, Portuguese: “

Item factor loadings ranged from 0.56 to 0.75, with questions 5 and 3 having the lowest loadings at 0.56 and 0.58, respectively. Average interitem covariances ranged from 0.17 to 0.21. One latent factor of depression explained 54.2% of the variance in PHQ-9 scores. The overall Cronbach’s alpha was 0.84, suggesting a good overall internal consistency of the PHQ-9 scale. Individually removing question 3 or question 5 did not result in an improvement in the overall Cronbach’s alpha. When considering the PHQ-2 only (Questions PHQ1 and PHQ2), the Cronbach’s alpha was 0.61 (see Table 3 for more information).

### Criterion validity of PHQ-9-MZ in screening for major depressive disorder

**Fig. 5:**
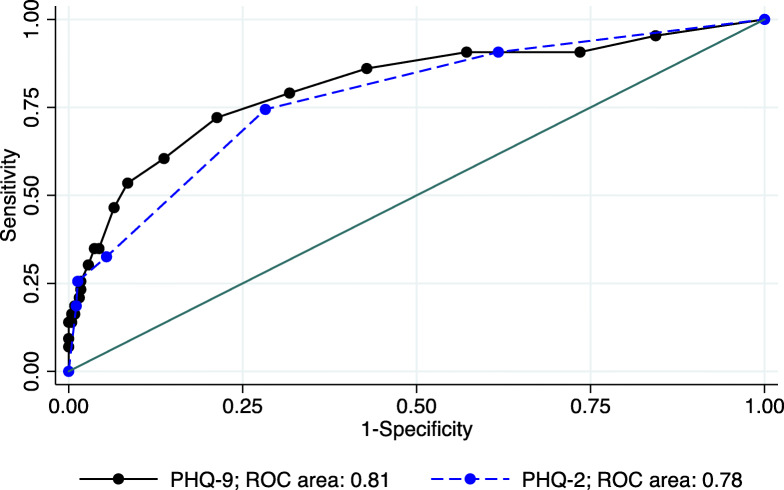
Receiver operating characteristic (ROC) curves for both the Patient Health Questionnaire 9-item (PHQ-9) and the Patient Health Questionnaire 2-item (PHQ-2) to detect major depressive disorder among primary care patients in Mozambique

The PHQ-9-MZ showed an acceptable AUROC of 0.81 (95% CI: 0.73, 0.89). The PHQ-2 achieved an AUROC of 0.78 (95% CI: 0.70, 0.85); (see Fig. 5). For the PHQ-9-MZ, Youden’s Index (Youden’s J) identified an optimal cut-point of ≥6, with a maximum value of sensitivity – (1-specificity) of 51%. However, for many applications, the low specificity at this cut-off may be undesirable. At this cut-point, the PHQ-9-MZ had a sensitivity of 72.1% and a specificity of 78.7% (see Table 4). The likelihood ratio positive was 3.4, with a diagnostic odds ratio of 9.5 and a positive predictive value of 0.24. With a cut-point of ≥9, the PHQ-9-MZ had a sensitivity of 46.5% and a specificity of 93.5%. The likelihood ratio positive was 7.1, with a diagnostic odds ratio of 12.5 and a positive predictive value of 0.40. With a cut-point of ≥11, the PHQ-9-MZ had a sensitivity of 34.9% and a specificity of 96.3%. The likelihood ratio positive was 9.4, with a diagnostic odds ratio of 14.0 and a positive predictive value of 0.47. In terms of the PHQ-2-MZ, Youden’s Index identified an optimal cut-point of ≥2, yielding a maximum value of sensitivity – (1-specificity) of 46%. At this cut-point, the PHQ-2-MZ had a sensitivity of 74.4% and a specificity of 71.7%. The likelihood ratio positive was 2.6, with a diagnostic odds ratio of 7.4 and a positive predictive value of 0.20. With a cut-point of ≥3, the PHQ-2-MZ had a sensitivity of 32.6% and a specificity of 94.6%. The likelihood ratio positive was 6.0, with a diagnostic odds ratio of 8.4 and a positive predictive value of 0.36.

**Table 4:** Performance of the Patient Health Questionnaire 9-item (PHQ-9) and Patient Health Questionnaire 2-item (PHQ-2) Screening Tools to Detect Major Depressive Disorder Among Primary Care Patients in Sofala, Mozambique

PHQ-9									PHQ-2							
Cut-point	Sens	Spec	% Classified	LR+	LR-	DOR	PPV	NPV	Sens	Spec	% Classified	LR+	LR-	DOR	PPV	NPV
≥0	100.0%	0.0%	8.6%	1.00					100.0%	0.0%	8.6%	1.00				
≥1	95.4%	15.7%	22.5%	1.13	0.30	3.80	0.10	0.97	90.7%	38.3%	42.7%	1.47	0.24	6.04	0.12	0.98
≥2	90.7%	26.5%	32.0%	1.23	0.35	3.52	0.10	0.97	**74.4%**	**71.7%**	**72.0%**	**2.63**	**0.36**	**7.38**	**0.20**	**0.97**
≥3	90.7%	42.8%	46.9%	1.59	0.22	7.30	0.13	0.98	**32.6%**	**94.6%**	**89.3%**	**5.99**	**0.71**	**8.40**	**0.36**	**0.94**
≥4	86.1%	57.2%	59.6%	2.01	0.24	8.23	0.16	0.98	25.6%	98.7%	92.5%	19.61	0.75	26.01	0.65	0.93
≥5	79.1%	68.3%	69.2%	2.49	0.31	8.13	0.19	0.97								
≥6	72.1%	78.7%	78.1%	3.38	0.35	9.54	0.24	0.97	18.6%	98.9%	92.1%	17.12	0.82	20.80	0.61	0.93
≥7	60.5%	86.3%	84.1%	4.41	0.46	9.64	0.29	0.96	0.0%	100.0%	91.5%		1.00			0.91
**≥8**	**53.5%**	**91.5%**	**88.3%**	**6.31**	**0.51**	**12.41**	**0.37**	**0.95**								
**≥9**	**46.5%**	**93.5%**	**89.5%**	**7.13**	**0.57**	**12.46**	**0.40**	**0.95**								
**≥10**	**34.9%**	**95.7%**	**90.5%**	**8.02**	**0.68**	**11.79**	**0.43**	**0.94**								
≥11	34.9%	96.3%	91.1%	9.44	0.68	13.96	0.47	0.94								
≥12	30.2%	97.2%	91.5%	10.70	0.72	14.90	0.50	0.94								
≥13	25.6%	98.3%	92.1%	14.71	0.76	19.42	0.58	0.93								
≥14	23.3%	98.3%	91.9%	13.37	0.78	17.12	0.56	0.93								
≥15	20.9%	98.5%	91.9%	13.75	0.80	17.13	0.56	0.93								
≥16	18.6%	99.1%	92.3%	21.40	0.82	26.06	0.67	0.93								
≥18	16.3%	99.1%	92.1%	18.72	0.84	22.17	0.64	0.93								
≥19	16.3%	99.4%	92.3%	24.96	0.84	29.62	0.70	0.93								
≥20	16.3%	99.6%	92.5%	37.44	0.84	44.53	0.78	0.93								
≥21	14.0%	99.6%	92.3%	32.09	0.86	37.14	0.75	0.93								
≥22	14.0%	99.8%	92.5%	64.19	0.86	74.44	0.86	0.93								
≥24	14.0%	100.0%	92.6%		0.86		1.00	0.93								
≥25	9.3%	100.0%	92.3%		0.91		1.00	0.92								
≥27	7.0%	100.0%	92.1%		0.93		1.00	0.92								
> 27	0.0%	100.0%	91.5%		1.00			0.91								
**Area Under ROC curve: 0.809 (95% CI: 0.731, 0.887)**									**Area Under ROC curve: 0.777 (95% CI: 0.703, 0.851)**							

Abbreviations: *LR+* Likelihood Ratio Positive, *LR-* Likelihood Ratio Negative, *DOR* Diagnostic Odds Ratio, *PPV* Positive Predictive Value, *NPV* Negative Predictive Value, *ROC* Receiver Operating Characteristic, *CI* Confidence Interval

## Discussion

This study aimed to test the validity and initial item response theory properties of the Mozambican-adapted PHQ-9-MZ among primary care patients attending Ministry of Health clinics in central Mozambique. Overall, we found the PHQ-9-MZ to have good internal consistency and the instrument performed well at discriminating between depressed and non-depressed individuals. Depending on programmatic needs balancing sensitivity and specificity, we recommend individuals use a cut-off of ≥9 on the PHQ-9-MZ, which resulted in a sensitivity of 46.5% and a specificity of 93.5%. The AUROC was maximized with a cut-off of ≥6, although the low specificity (78.1%) at this cut-off would likely be undesirable for most applications.

The PHQ-2-MZ discriminated well, with a cut-off that maximizes AUROC of ≥2. However, again, given the low specificity at this cut-off, the PHQ-2-MZ might be best used as an initial screener, with the PHQ-9-MZ applied to those screening positive. For rapid screening of patients with a higher specificity, a cut-off of ≥3 could be used with the PHQ-2-MZ, although the low sensitivity at this cut-off might be undesirable (32.6%).

A meta-analysis published in 2012 of PHQ-9 validation studies has shown that cut-off scores between 8 and 11 are optimal for screening for depression across various clinical settings. Yet, since this study was published, there have been a number of PHQ-9 validation studies conducted in the Sub-Saharan African context. Two recent studies have identified a similar cut-off of ≥9 as optimal [34]. For example, a validation study in Dar es Salaam, Tanzania revealed an optimal cut-off score of 9 with a sensitivity of 78% and specificity of 87%, with a similar internal consistency to our study (α = 0.83) and slightly higher AUROC (0.87) [21]. Another validation study of the PHQ-9 in South Africa among patients with chronic conditions revealed an optimal cut-off score of ≥9, with a sensitivity of 49%, a specificity of 94% and reasonable internal consistency (α = 0.76) [20]. Alternatively, a study in primary healthcare in Zimbabwe revealed an optimal cut-off score of ≥11, with similar internal consistency (α = 0.84), a sensitivity of 85%, and specificity of 69%, against a SCID gold standard [35]. In a study of high HIV burden PHC population in Johannesburg, South Africa, an optimal cut-off score of ≥10 revealed a sensitivity of 79% and specificity of 83% and a high AUROC (0.88) [36], similar to other settings outside of sub-Saharan Africa [37]. Another study performed in Cameroon among ﻿HIV-infected patients, revealed a very low sensitivity of 27% and specificity of 94% when using a cut-off score of ≥10 [38]. The authors suggest that the poor performance of the PHQ-9 in Cameroon may be due to cultural factors, low literacy, or a low prevalence of depression in the surrounding communities. Thus, with the exception of Cameroon, our findings are broadly in line with similar PHQ-9 validation studies conducted in Sub-Saharan Africa suggesting good performance with PHQ-9 cut-off scores between 9 and 11.

We propose several cut points of PHQ-9-MZ, which could be used depending on the needs of the population being screened and the capacity of the immediate healthcare environment. A cut-off score of ≥6 maximizes the AUROC, but the low specificity could lead to many false positives. This may be appropriate if screening is focusing on a population with high prevalence of depression, but many false positives in a population with a low prevalence of depression could unnecessarily increase the burden on a health system that already faces resources shortages for mental healthcare. We recommend using a PHQ-9-MZ cut-off score between ≥8 and ≥ 11. We believe these scores appropriately balance high sensitivity and high specificity, which may be optimal for screening in Mozambican primary care settings.

One alternative to applying the PHQ-9 and PHQ-2 separately is to apply the PHQ-2-MZ first, and then apply the PHQ-9-MZ to patients screening positive. Thus, one could begin with a rapid high sensitivity/low specificity test (PHQ-2-MZ), followed by a longer second test with high specificity (PHQ-9-MZ). This maximizes the probability that those initially screened as false positives can be correctly identified as negative [39]. Given our study results, individuals could use the PHQ-2-MZ with a cut-off score of ≥2 for initial screening, followed by the PHQ-9-MZ for those with initial positive result. The PHQ-2 has been recommended in many studies as an initial screening tool before applying the full PHQ-9 [40, 41].

A further finding from our study was that the question *“sleeping too much or too little”* (question 3) and *“loss of appetite”* (question 5) had poor discrimination across depressive symptoms compared to the other PHQ-9-MZ items. Unfortunately, few existing PHQ-9 validation studies in Sub-Saharan Africa have conducted item response theory analyses to examine the performance of individual PHQ-9 questions [20, 35, 36, 38]. One existing study from Tanzania reported individual factor loadings, showing that the *“loss of appetite”* item represented a separate factor of “appetite” separate from the factor of depressed mood / self-harm [21]. This factor of “appetite” explained only 5% of the total variance, whereas the factor of depressed mood / self-harm explained 76% of the variance. In addition, the *“sleeping too much or too little”* question was included in a separate factor described as physiological symptoms that explained 13% of the variance. These findings corroborate our findings in the present study that the appetite and sleep related questions had the lowest factor loadings for the overall single latent factor of depression. One reason for the poor discrimination of the sleep and appetite questions may be that these symptoms are not as closely related to depressive symptoms in Mozambique and Tanzania when compared to other contexts, such as the United States. In a setting like Mozambique where a significant proportion of the population is food insecure, appetite changes may be more related to availability of food and economic conditions than depressive symptoms. However, it is possible that these questions were not appropriately adapted to Mozambican Portuguese idioms, and that these questions were misunderstood or unclear. Future validation studies targeting the PHQ-9 and other mental health screening tools should examine item response theory properties and examine the performance of individual questions. Further research could consider how to improve the PHQ-9 for use in Sub-Saharan African settings and consider replacing, reformulating, or improving these questions to better fit the local context. Analyses of individual items is essential to understanding the performance and help improve the fit of screening tools to the local context. For example, the PHQ-9 discriminated poorly for depression in a previous study in Cameroon [38], yet the researchers did not conduct item response theory analyses which would have helped analyze whether there was heterogeneity in how individual items performed in this context. These analyses can help to improve the cultural fit of screening tools, improve adaptation to local idioms, or inform replacing poorly - performing questions with concepts more culturally - linked to depressive symptoms across diverse contexts.

Our study had several important limitations. First, we relied on PHQ-9-MZ and MINI 5.0-MZ responses emanating from patients attending three public-sector clinics in urban and peri-urban contexts in Sofala Province, Mozambique. It may be that our instrument validation results from this context will not generalize to other areas in Mozambique and especially in rural areas; we suggest follow-up replication studies in rural areas and across other regions of Mozambique. Second, due to the low literacy in this population, we relied on an interviewer-administered PHQ-9-MZ rather than a self-administered instrument. Thus, it is unclear how our results may extend to individuals who may self-administer the instrument. Last, this instrument was developed and validated in Mozambican Portuguese, rather than any of the common local indigenous languages in Central Mozambique such as Sena, Ndau, or Shona. Future work could develop local idiomatic adaptations of the PHQ-9 into common local indigenous languages to facilitate understanding, especially in rural areas.

Notwithstanding these limitations, our study had a number of strengths. This is the first study to validate the PHQ-9 for use in Mozambique. We recruited a random sample of patients attending multiple primary care services (outpatient, pre- and post-partum) and employed a team of bilingual experts and laypeople to engage in a multistage process of instrument adaptation prior to instrument implementation. In addition, we conducted modern item response theory analyses to contribute to the literature on individual PHQ-9 item performance and the improvement of depression measurement in Sub-Saharan Africa.

## Conclusions

In summary, we found the PHQ-9-MZ and PHQ-2-MZ to be valid and effective screening tools for depression in primary health care settings in Mozambique. For most applications we recommend using a cut-off score of ≥9 on the PHQ-9-MZ to maximize sensitivity while minimizing false positives for primary care depression screening. However, we recommend PHQ-9-MZ users to examine the results of this study and select a cut-off score that meets their needs to balance false positives and false negatives. For rapid screening, the PHQ-2-MZ can be implemented with cut-offs of ≥2 or ≥ 3 – although the lower cut-off will result in high rates of false positives and the higher cut-off will result in high rates of false negatives. An alternative framework could be to apply the PHQ-2-MZ with the high sensitivity cut-off of ≥2 and then follow-up these positive patients with the PHQ-9-MZ at a cut-off with a higher specificity. Overall, the use of valid screening tools to screen for primary care in Mozambique is urgently needed given the large and persistent treatment gap for depression. Further work could focus on developing depression screening tools in local indigenous languages, and potentially improving the PHQ-9-MZ to more effectively represent depression symptoms common in Mozambique.

## Data Availability

The datasets used and/or analyzed during the current study are not publicly available due to Mozambican IRB regulations and lack of previous approval to share data publicly. The datasets used and/or analyzed during the current study can be made available through a data-sharing agreement with the corresponding author on reasonable request.

## Abbreviations

AUROC: Area Under Receiver Operating Characteristic
CI: Confidence Interval
DOR: Diagnostic Odds Ratio
GBD: Global Burden Disorder
IRT: Item Theory Response
LMICs: Low - and Middle - Income Countries
LR-: Likelihood Ratio Negative
LR +: Likelihood Ratio Positive
MDD: Major Depressive Disorder
MINI: Mini International Neuropsychiatric Interview
MISAU: Ministry of Health, Mozambique
MZ: Mozambique
MZN: Currency of Mozambique
NPV: Negative Predictive Value
PHQ-2MZ: Patient Health Questionnaire 2, Mozambique Version
PHQ-9MZ: Patient Health Questionnaire 9, Mozambique Version
PPV: Positive Predictive Value
ROC: Receiver Operating Characteristic
Sens: Sensitivity
Spec: Specificity
TPMH: Technician of Psychiatry and Mental Health
WHO: World Health Organization
YLD: Years Lived with Disability
